# Effect of physical activity on pulse wave velocity in elderly subjects with normal glucose, prediabetes or Type 2 Diabetes

**DOI:** 10.1038/s41598-018-25755-4

**Published:** 2018-05-23

**Authors:** Erja Metsämarttila, Enrique Rodilla, Jari Jokelainen, Sauli Herrala, Juhani Leppäluoto, Sirkka Keinänen-Kiukaanniemi, Karl-Heinz Herzig

**Affiliations:** 1 Research Unit of Biomedicine, and Biocenter of Oulu, Oulu University, 90014 Oulu, Finland; 2 Hypertension Clinic, Internal Medicine, Hospital de Sagunto, Valencia, Spain; 3 Center for Life Course Epidemiology and Systems Medicine, Faculty of Medicine, University of Oulu, 90014 Oulu, Finland; 4 Oulu University Hospital, Unit of General Practice, and Health Center of Oulu, Oulu, Finland; 5 Department of Gastroenterology and Metabolism, Poznan University of Medical Sciences, Poznan, Poland; 6 Medical Research Center (MRC) and University Hospital, Oulu, Finland; 7 Universidad Cardenal Herrera-CEU, CEU Universities, Valencia, Spain

## Abstract

Carotid-femoral pulse wave velocity ((cf)PWV) is a measure of arterial stiffness, predicting cardiovascular disease. We hypothesized that the amount of physical activity (PA) is correlated with reduced arterial stiffness in Type 2 diabetic (T2D) subjects. 570 subjects from the 1945 Oulu birth cohort were included in the analysis. (cf)PWV was determined by a non-invasive applanation tonometry. Oral glucose tolerance test was performed and LDL and HDL cholesterol analyzed. PA was registered daily with a wrist-worn acceleration meter for two weeks. (cf)PWV values in subjects with impaired glucose metabolism (IGM) and T2D were higher than in normal glycemic subjects (P < 0.001). PA, fasting and 2 h glucose and HbA1c correlated significantly with (cf)PWV, but HDL or LDL cholesterol did not. The 2 h glucose, heart rate and alcohol consumption in T2D subjects had independent effects on (cf)PWV in multiple regression analysis. T2D and IGM were significantly associated to (cf)PWV. Interestingly, lipids did not have an additional effect on (cf)PWV. Subjects walking more than 10 000 steps/day had 0.2 m/s lower (cf)PWV than those walking less than 6000 steps/day. Presence of T2D, elevated heart rate and alcohol consumption in males were associated with increased aortic stiffening in elderly subjects.

## Introduction

The incidence of type 2 diabetes (T2D) has been steadily increasing since 1980 and a reverse of this trend seems not to be likely^1^. Hyperglycemia is one of the major risk factor for cardiovascular diseases and damages blood vessel endothelium^2^, especially with age.

Aging stiffens the arterial walls with increased amounts of collagen in the arterial wall. This arterial stiffness has been suggested as a biomarker in the prediction of cardiovascular diseases^3–5^. Stiffened arteries result in an increase of blood pressure in systole and decrease in diastole by the earlier return of the reflected pressure wave hindering coronary blood flow^6–10^. The stiffening of the aorta can be noninvasively measured by tonography of the pulse wave traveling between the carotid and femoral artery in meters (m) divided by time (seconds (s)); this has been termed carotid-femoral pulse wave velocity ((cf)PWV)^6^. Previous studies have demonstrated protective effects by exercise^11–14^.

T2D patients have been shown to have higher (cf)PWV compared with healthy peers^15–19^. Agnoletti *et al*. found this effect only in subjects of 60 years and older but not in younger subjects^20^. Insulin directly affects the arterial structure by increasing collagen synthesis and creating hyperplasia and hypertrophy in arterial smooth muscle cells^21,22^.

We hypothesized that the amount of physical activity (PA) is correlated with reduced arterial stiffness in Type 2 diabetic (T2D) subjects. We examined associations between (cf)PWV, normal & impaired glucose, lipid metabolism and life style factors in our cohort subjects born in 1945 living in the City of Oulu, Finland.

## Methods

### Study population

**Figure 1:**
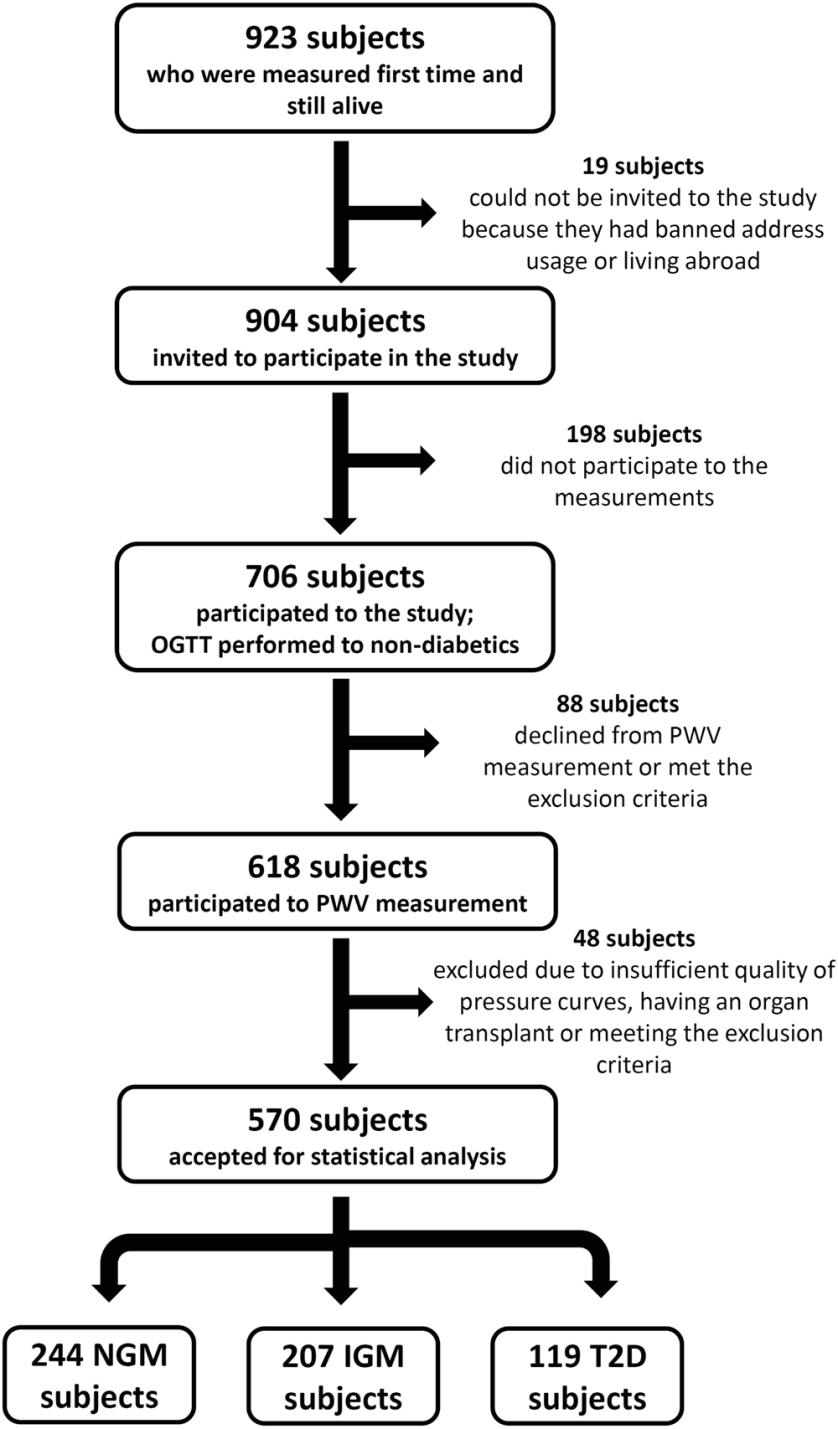
Flow chart of the study participants.

The study population consisted of the all the subjects born in 1945 in the City of Oulu, Finland (Oulu 1945 birth cohort)^23^. 904 individuals from the original 1945 cohort were invited for a follow-up study during the years 2013–2015 (Fig. 1). Exclusion criteria were BMI over 40 kg/m^2^ or irregular heart rate know to disturb (cf)PWV measurement. The protocol was approved by the local institutional ethics committee (Northern Ostrobothnia Hospital District) and in compliance with national legislation and the Declaration of Helsinki. All subjects gave their informed written consent.

### Measurements

Habitual physical activity of the participants was measured objectively with a wrist-worn acceleration meter (Polar Active, Polar Electro, Finland^24^. The participants wore the activity meter during the wakeful time for two weeks. We utilized a non-invasive applanation tonometry SphygmoCor Cardiovascular Management System (Version 8.2, AtCor Medical, Australia) for measuring pulse wave velocity. The SphygmoCor measurements were carried out by the same observer in every subject to avoid any inter-observer variability. (cf)PWV measurement was conducted in a supine position. The distance between the carotid measurement site and the suprasternal notch was measured by tape measure, while the distance between the femoral measurement site and the supra sternal notch was determined with a specially designed caliper. We adjusted the raw (cf)PWV data to the mean arterial blood pressure according to the “The Reference Values for Arterial Stiffness’ Collaboration” and age^25^. BP was determined with an automated blood pressure monitor (Omron M3, Omron Healthcare Europe B.V., Netherlands).

### Study protocol

The participants performed up to three study visits. During the first visit after an overnight fast a bioelectrical impedance analysis (InBody 720, InBody, Seoul, Korea) was conducted and blood samples taken. During the second visit an oral glucose tolerance test (OGTT) was performed to those who were not diagnosed with T2D. Subjects were diagnosed with T2D, if they had diabetes medication or if in glucose tolerance tests either the fasting value was equal or above 7 mmol/l or the two-hour-value was above 11.1 mmol/l. Subjects were categorized as impaired glucose metabolism (IGM), if they had impaired fasting glucose (fasting glucose level 6.1 to 6.9 mmol/l) or impaired glucose tolerance (two-hour glucose level 7.8 to 11.0 mmol/l). The participants filled questionnaires regarding their life style, mental status and perceived health. Smoking history and present status was inquired by questionnaires and results categorized into 3 groups: non-smokers, former smokers (>6 months) and smokers. Alcohol consumption was evaluated by frequency, type and amount and translated into grams alcohol/day. At the end of the second visit the participants were given an activity meter (Polar Active, Polar Electro, Finland).

During the third visit, pulse wave velocity measurements were conducted. The participants were instructed to abstain from alcohol for 12 hours, from caffeine and tobacco for four hours and from food for six hours prior to the measurement. A light meal was allowed if complete fasting was not possible e.g. because of diabetes medication. The subject was asked to take a supine position and (cf)PWV measurement was conducted on their left side. The time interval between the OGTT and the (cf)PWV measurements was less than 9 months.

### Statistics

Values reported are mean ± SD for continuous variables and percentages for categorical variables. Daily steps were divided into quartiles. P-value of <0.05 was considered significant. Kruskal-Wallis test was used for comparison of continuous variables between groups. Differences in frequency were tested by chi-square test. Spearman’s rho was used to compare continuous variables. Multiple regression analysis was performed to assess linear associations between (cf)PWV and metabolic status. We adjusted our multivariate regression analyses for potential confounding variables such as gender, alcohol, BMI, number of daily steps, smoking, HDL cholesterol, and triglycerides as previously described^26–28^. To correct for multiple testing the false discovery rate (FDR) was controlled at 0.25 by using the Benjamini-Hochberg method^29^. Statistical analyses were done on R version 3.2.3 (https://www.R-project.org/) and IBM SPSS Statistics 21.

## Results

Compete data sets from 570 subjects (338 female and 232 male) were available for statistical evaluation (Fig. 1). Anthropological and biochemical characteristics are listed in Table 1 according to their glucose status. Obesity was two times higher in IGM and T2D than in normal glucose metabolism (NGM) subjects (p < 0.001). There were no differences in the numbers of current smokers between the groups, but IGM and T2D subjects consumed about 2 times more alcohol than NGM subjects (p < 0.001). Systolic blood pressure was higher in T2D subjects than in the others (p < 0.004). (cf)PWV was higher in IGM and T2D than in NGM subjects, while the number of daily steps were lower in the NGM than in the IGM and T2D subjects (p < 0.001). The incidence of hypertension and coronary arterial disease (CAD) was increased in IGM and T2D than in NGM subjects, reflected also by use of hypertensive medication, anti-lipid drugs and nitrates (p < 0.001). LDL and HDL cholesterol levels were higher in NGM than in IGM and T2D subjects (p < 0.007 and p < 0.001, respectively).

**Table 1:** Anthropological and biochemical characteristics of the study population. Means ± standard deviations or numbers of subjects.

	Total	NGM	IGM	T2D	P value
No of subjects	570	244	207	119	
Gender (m)	232 (41%)	88 (36%)	82 (40%)	62 (52%)	0.014
Age (years)	68.5 ± 0.6	68.5 ± 0.7	68.5 ±0 .6	68.5 ± 0.6	0.507
Weight (kg)	75.0 ± 13.6	71.4 ± 12.5	75.0 ± 12.6	82.3 ± 14.7	<0.001
Height (m)	166 ± 8	166 ± 8	165 ± 8	166 ± 8	0.362
BMI (kg/m^2^)	27.3 ± 4.0	25.9 ± 3.4	27.5 ± 3.9	29.7 ± 4.2	<0.001
Obese subjects	136 (24%)	25 (10%)	52 (25%)	59 (50%)	<0.001
Alcohol (g/d)	1.7 ± 4.5	1.1 ± 1.8	2.4 ± 6.6	2.1 ± 3.7	0.273
Self-reported smoking					0.001
Current	67 (12%)	25 (10%)	20 (10%)	22 (19%)	
Former	185 (33%)	67 (27%)	69 (33%)	49 (42%)	
Non-smokers	308 (54%)	149 (61%)	113 (55%)	46 (39%)	
SP (mmHg)	142 ± 18	141 ± 18	143 ± 17	144 ± 19	0.188
DP (mmHg)	78 ± 8	77 ± 8	79 ± 8	78 ± 8	0.158
No of HT	235 (41%)	70 (29%)	96 (46%)	69 (58%)	<0.001
No of CAD	60 (11%)	14 (6%)	19 (9%)	27 (23%)	<0.001
Antihypertensive medication	249 (50%)	80 (37%)	94 (55%)	75 (67%)	<0.001
Lipid lowering medication	167 (33%)	51 (/24%)	65 (38%)	51 (46%)	<0.001
Diabetes medication	62 (11%)			62 (11%)	<0.001
Daily steps	8877 ± 3683	9419 ± 3629	8707 ± 3481	8062 ± 3977	0.001
Fasting glucose (mmol/l)	5.6 ± 0.6	5.4 ± 0.4	5.7 ± 0.5	6.2 ± 0.8	<0.001
2 h glucose (mmol/l)	6.9 ± 1.9	5.6 ± 1.1	7.6 ± 1.4	9.3 ± 2.4	<0.001
HbA1c (mmol/l)	40.5 ± 5.2	38.7 ± 3.5	40.1 ± 3.9	44.9 ± 7.1	<0.001
Total cholesterol (mmol/l)	5.4 ± 1.2	5.5 ± 1.2	5.4 ± 1.1	5.0 ± 1.4	<0.001
HDL cholesterol (mmol/l)	1.7 ± 0.5	1.8 ± 0.5	1.6 ± 0.4	1.5 ± 0.4	<0.001
LDL cholesterol (mmol/l)	3.4 ± 1.1	3.5 ± 1.1	3.4 ± 1.0	3.2 ± 1.3	0.007
(cf)PWV (m/s)	8.8 ± 1.8	8.3 ± 1.7	9.0 ± 1.9	9.4 ± 1.7	<0.001
MBP adj. (cf)PWV (m/s)	8.8 ± 1.6	8.4 ± 1.5	9.0 ± 1.8	9.3 ± 1.5	<0.001

P values calculated with Kruskal-Wallis rank sum test or chi-square test between the groups. The Total column is not part of the calculations.

NGM, normal glucose metabolism; IGM, impaired glucose metabolism; T2D, type 2 diabetes; BMI, body mass index; SP, brachial systolic blood pressure; DP, brachial diastolic blood pressure; HT, hypertension; No, number of; CAD, coronary artery disease; HbA1c, glycated hemoglobin; HDL, high-density lipoprotein; LDL, low-density lipoprotein; (cf)PWV, carotid-femoral pulse wave velocity; MBP, mean blood pressure.

Results of the multiple linear regression analysis are presented in Table 2. (cf)PWV was adjusted for different parameters in four models. Model 1 was adjusted for metabolic status, Model 2 for the metabolic status and HR and Model 3 further for alcohol consumption, obesity, sex, and number of daily steps. The positive association between (cf)PWV and 2 h glucose in T2D and IGM subjects remained significant after the adjustments in Models 1 and 2 and between (cf)PWV and 2 h glucose in Model 3. In addition, heart rate remained significant. (cf)PWV associated significantly with alcohol consumption but not with obesity, gender, smoking, daily steps, HDL cholesterol or lipids. In Model 4, addition of hypertension medication did not have an effect on (cf)PWV. Exclusion of the subjects with a systolic blood pressure over 160 mmHg did not change the results of Model 4.

**Table 2:** Multiple regression analysis of the cohort. Dependent variable (β, beta coefficient; CI confidence intervals).

	Model 1	Model 2	Model 3	Model 4
	(1) n = 570	(2) n = 570	(3) n = 476	(4) n = 476
IGM	0.548 (0.252, 0.845) p = 0.0004***	0.457 (0.162, 0.751) p = 0.003**	0.221 (−0.108, 0.550) p = 0.189	0.203 (−0.127, 0.533) p = 0.230
T2D	0.920 (0.569, 1.271) p = 0.00000***	0.840 (0.491, 1.189) p = 0.00001***	0.626 (0.211, 1.042) p = 0.004**	0.586 (0.166, 1.007) p = 0.007**
HR		0.038 (0.022, 0.055) p = 0.00001***	0.038 (0.019, 0.057) p = 0.0001***	0.039 (0.020, 0.058) p = 0.0001***
Gender		0.107 (−0.159, 0.374) p = 0.431	0.026 (−0.322, 0.374) p = 0.885	0.030 (−0.318, 0.378) p = 0.866
Alcohol consumption (g alcohol/day)			0.067 (0.018, 0.116) p = 0.009**	0.069 (0.020, 0.119) p = 0.007**
Obesity (BMI)			0.341 (−0.027, 0.710) p = 0.070	0.318 (−0.052, 0.689) p = 0.093
PA (1000 steps)			−0.016 (−0.056, 0.025) p = 0.445	−0.014 (−0.055, 0.026) p = 0.492
Smoking (former)			−0.222 (−0.553, 0.109) p = 0.191	−0.231 (−0.562, 0.100) p = 0.173
Smoking (current)			−0.383 (−0.855, 0.089) p = 0.113	−0.388 (−0.860, 0.084) p = 0.108
HDL cholesterol (mmol/l)			−0.107 (−0.480, 0.266) p = 0.575	−0.095 (−0.469, 0.279) p = 0.618
Triglycerides (mmol/l)			0.029 (−0.142, 0.200) p = 0.743	0.032 (−0.139, 0.203) p = 0.714
Anti –Hypertension medication				0.172 (−0.123, 0.467) p = 0.253

Note: ^*^p < 0.05; ^**^p < 0.01; ^***^p < 0.001.

IGM, impaired glucose metabolism; T2D, type 2 diabetes; HR, heart rate; PA, physical activity, Model 1 includes IGM and T2D; Model 2 includes Model 1 variables plus HR, and Model 3 includes variables from Model 1 & 2 and alcohol consumption, obesity; Model 4 included variables from Model 1–3 and anti-hypertensive medication.

**Figure 2:**
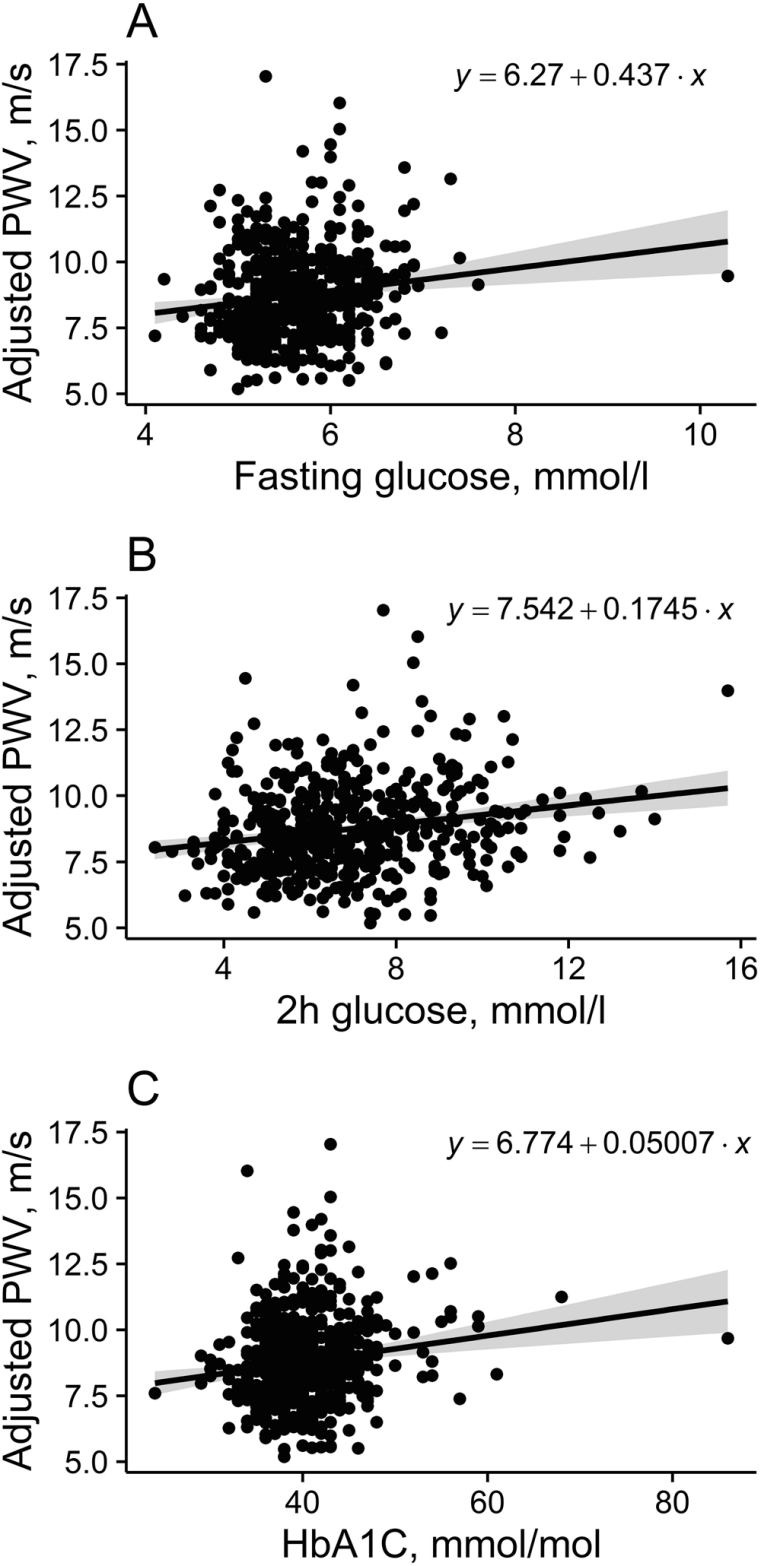
Associations between pulse wave velocity ((cf)PWV) and fasting glucose (**A**) and 2 h glucose (**B**) and glycated hemoglobin (HbA1c) (**C**). Shaded area represents 95% CIs around the regression line.

**Figure 3:**
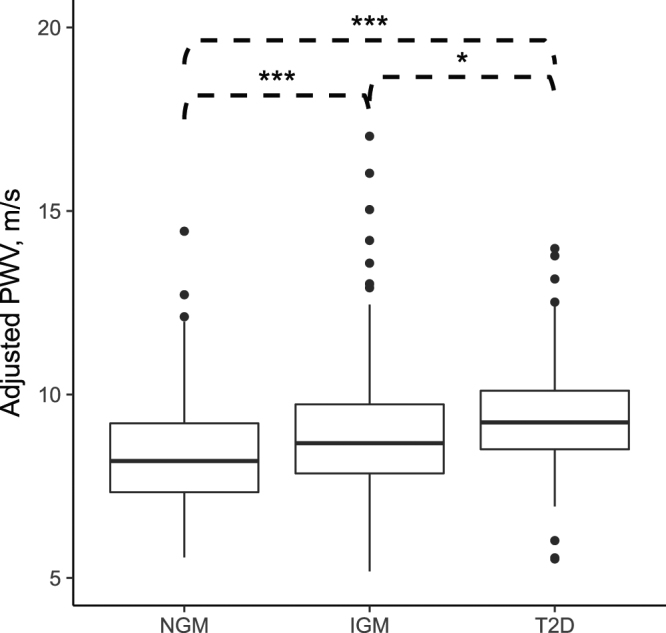
Boxplot presentation of pulse wave velocity ((cf)PWV) in normal glucose metabolism (NGM), impaired glucose metabolism (IGM) and type 2 diabetes (T2D). The horizontal lines represent the median values, the boxes interquartile ranges, error bars 95*%* confidence intervals. ***P < 0.001, *P < 0.05.

Linear associations between (cf)PWV and fasting and 2 h glucose and HbA1c concentrations are shown in Fig. 2. Fasting and 2 h glucose and HbA1c all correlated positively with (cf)PWV (r = 0.15, 0.20 and 0.16, respectively, P < 0.001 for all), but the correlation was rather weak, suggesting that other factors contribute as well.

(cf)PWV of subjects with normal or impaired glucose metabolism or T2D are shown in Table 2 and Fig. 3. IGM subjects had 0.55 m/s (average 8.96 ± 1.77 m/s) and T2D subjects had 0.92 m/s (average 9.33 ± 1.50 m/s) higher (cf)PWV than NGM subjects (average 8.41 ± 1.49 m/s). (cf)PWV values of IGM and T2D subjects differed clearly from the values of NGM subjects (P < 0.001) and less between IGM and T2D subjects (P < 0.05).

**Figure 4:**
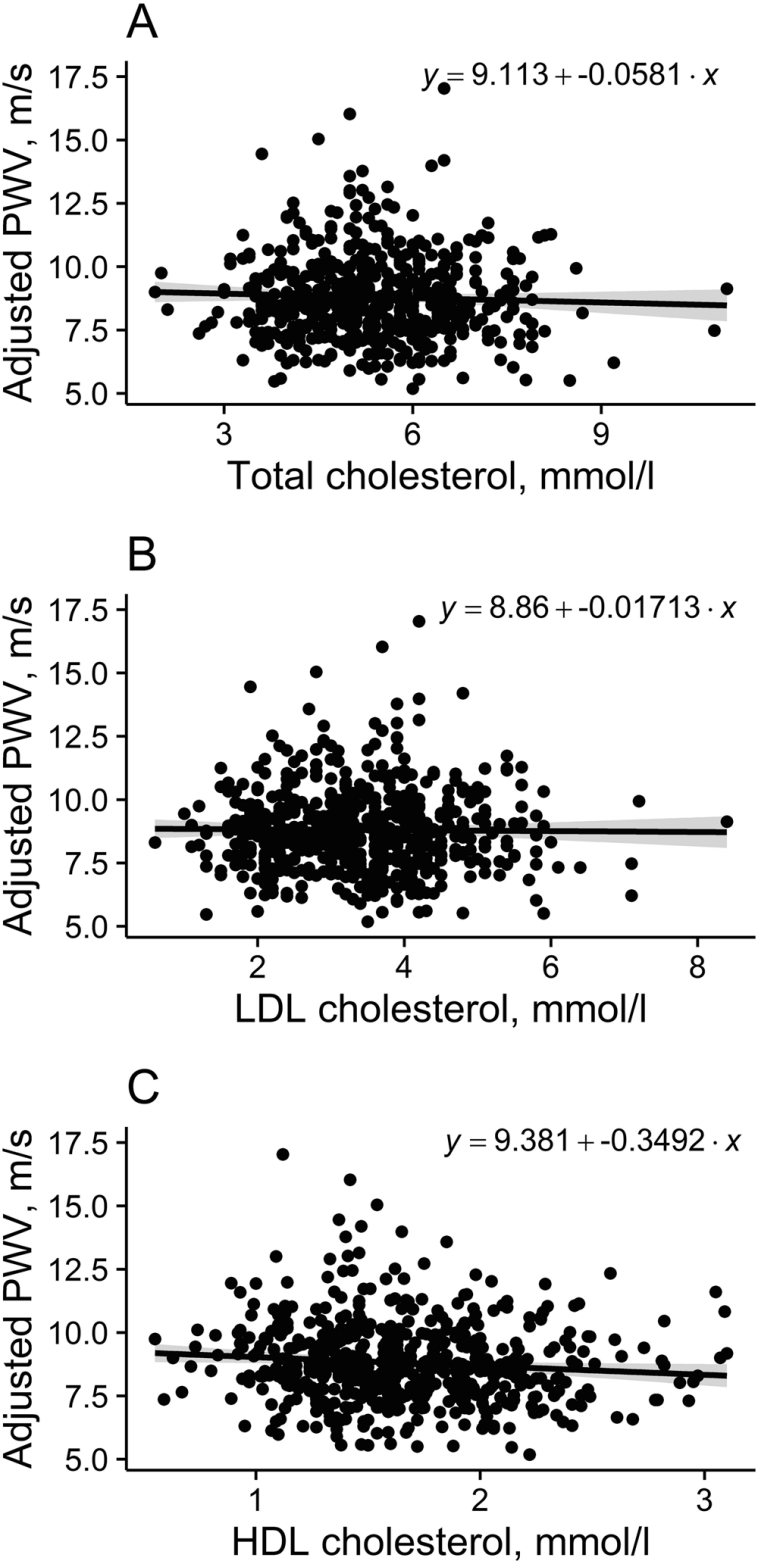
Associations between pulse wave velocities ((cf)PWV) and total (**A**), LDL (**B**) and HDL (**C**) cholesterol. Shaded area represents 95% CIs around the regression line.

Associations of (cf)PWV with total, LDL and HDL plasma cholesterol are shown in Fig. 4. There was no correlation between (cf)PWV and total nor LDL cholesterol (r = −0.03 and −0.01, respectively, P > 0.1 for both), but a negative correlation existed between (cf)PWV and HDL cholesterol (r = −0.11, P < 0.05).

**Figure 5:**
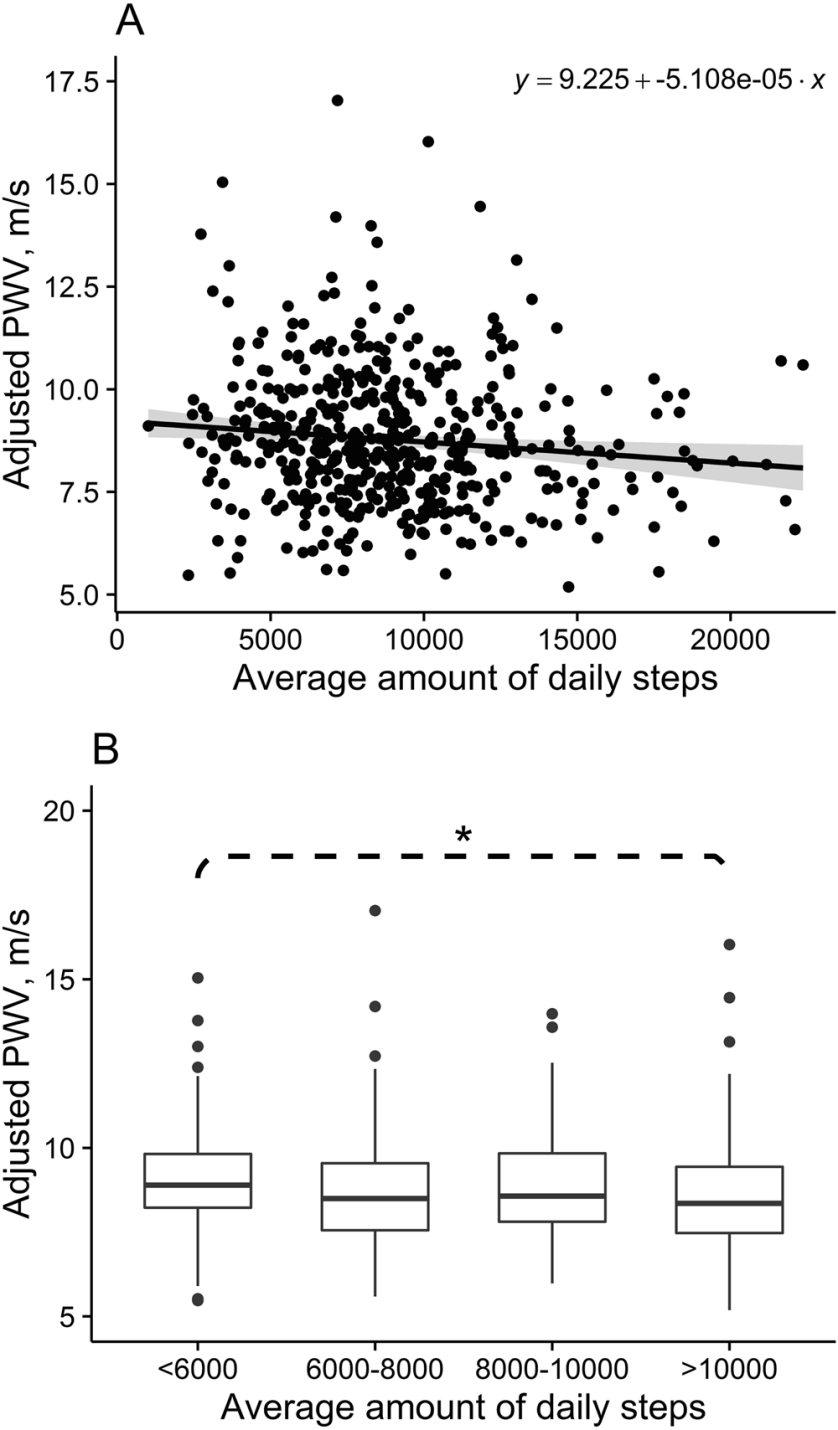
(**A**) Linear correlation between physical activity (steps/day) and pulse wave velocity ((cf)PWV). Shaded area represents 95% CIs around the regression line (**B**) Differences in (cf)PWV in physical activity quartiles. The horizontal lines represent the median values, the boxes interquartile ranges, error bars 95*%* confidence intervals. Outside values are presented. *P < 0.05.

We analyzed the association of (cf)PWV and daily steps and observed that every increment of 1000 steps per day was associated with decrease of 0.05 m/s in (cf)PWV. In order to study closer the associations between (cf)PWV and daily steps, we compared differences between quartiles of the daily step numbers. There was a significant difference between the first and fourth quartile (P < 0.05) representing steps less than 6000 and over 10000 steps per day (Fig. 5).

## Discussion

Previous studies have shown that in middle-aged and older subjects arterial stiffening is positively associated with glucose metabolism^18,30–32^. In our cohort of aged subjects (68.5 ± 0.5 yrs) we found the lowest (cf)PWV in NGM and the highest in T2D. Taniwaki *et al*. reported that T2D and high blood pressure were associated with increased (cf)PWV in middle-aged subjects with a positive correlation between carotid intima thickness and (cf)PWV in T2D compared to healthy subjects^15^. The authors suggested that the increased blood pressure might be the cause for the arterial stiffening.

In our study, we observed an increased (cf)PWV in IGM subjects compared to NGM with was further increased in T2D, suggesting that the increased glucose level is a significant factor in the development of arterial stiffening. (cf)PWV was significantly associated with fasting, 2 h glucose, HbA1c and obesity (P < 0.001 for all). The increase of 1 mmol/l in fasting glucose raised (cf)PWV by 0.4 m/s and an increase of 1 mmol/l in 2 h glucose raised (cf)PWV by 0.2 m/s. In our multiple regression analysis after adjusting to confounding factors HR, alcohol consumption and 2 h  glucose were still significantly associated with (cf)PWV (Table 2).

Schram *et al*. suggested that hyperglycemia induces qualitative and quantitative changes in elastin and collagen of the arterial walls^18^. Hyperglycemia leads to the formation of advanced glycation end products, which deteriorate the function of the arterial walls and disturbs cell function with receptor and nonreceptor pathways^33^. Wang *et al*.^34^ showed that accumulation of stabilized glycated proteins over a lifetime can contribute to increased vascular stiffness, endothelial dysfunction, and inflammation. Similarly, McEniery *et al*.^35^ found in a 20-year follow-up in 825 men of the Caerphilly Prospective Study that blood pressure and inflammation were more important predictors of aortic stiffness than other traditional cardiovascular risk factors. Importantly, changes in (cf)PWV might be detected before those in extracellular matrix turnover and fibrosis like markers (e.g. matrix metalloproteinases - MMP-2, MMP-9)^36^. The clinical relevance of this would be to encourage the subjects with higher PWV to undergo life style changes. Nordstrand and colleagues found that a moderate caloric restriction combined with aerobic physical exercise could significantly reduce arterial stiffness in morbidly obese individuals after a 7 weeks of intervention^37^. Dernellis *et al*. reported that aortic stiffness is an independent predictor of progression to hypertension in nonhypertensive individuals^38^.

The relationship between (cf)PWV and cholesterol is still unclear. Previous studies have found in adults below 60 years significant associations between (cf)PWV and total, LDL and/or HDL cholesterol^39–47^, whereas other studies did not^6,48–51^. Our results using univariate regression analysis showed no correlation between (cf)PWV and HDL and LDL cholesterol, indicating that cholesterol might have a lesser role than glucose levels in the stiffening of the arterial walls in these elderly subjects.

Jennersjö and colleagues reported that for every 1000 steps per day (cf)PWV was reduced by 0.103 m/s during a four-year-follow-up in middle-aged T2D subjects^11^. Moderate-to-vigorous physical activity was negatively associated with arterial stiffness in their middle-aged ambulatory community-dwelling subjects^52^. In their study the compliance with exercise guidelines was associated with 1% lower (cf)PWV which corresponds to arterial stiffness values of 1.8 years younger individuals. In our analysis we observed that for every 1000 steps more per day, there was a decrease of 0.05 m/s in (cf)PWV as 1000 step equals 10 minutes of walking at moderate pace^53^. Walking more than 10000 steps/day as our most active subjects did, the mean reduction in arterial stiffness should be 0.5 m/s, which is close to the difference we observed between NGM and IGM subjects.

Alcohol consumption has been shown to have a linear association with (cf)PWV. The light-to-moderate drinkers had lower (cf)PWV than non- or heavy drinkers^26,27^. In our subject the alcohol consumption was significantly and positively related to (cf)PWV. The IGM and T2D subjects used on average 2.1–2.4 g alcohol/day. In the multiple regression analysis there was a positive association between (cf)PWV and alcohol consumption of more than 6.1 g alcohol daily. Our findings suggest that alcohol consumption is a strong independent risk for cardiovascular diseases, since doses over 6 g/day were associated with arterial stiffening.

Our study is limited by the cross-sectional analysis of investigating only one age group in Northern Finland, but our strengths are the lack of ethnic differences in the study population and the performance of an OGTT in all subjects who were not previously diagnosed with T2D. PA was recorded objectively by an activity meter for 2 weeks. Larger cohorts might detect additional factors affecting aortic stiffening.

## Conclusions

We observed that aortic stiffness was associated with IGM and T2D but this association was reduced in subjects with elevated physical activity over 10000 steps/day. Cholesterol did not have an added effect on (cf)PWV in our elderly cohort. The changes in (cf)PWV seem to appear before changes in extracellular matrix turnover and fibrosis like markers^36^ giving the opportunity for clinical intervention. This would need to be tested in future interventions.
